# A T231E Mutant that Mimics Pathologic Phosphorylation of Tau in Alzheimer’s disease Causes Activation of the Mitochondrial Unfolded Protein Response in* C. elegans* touch neurons

**DOI:** 10.17912/micropub.biology.000306

**Published:** 2020-09-09

**Authors:** Sanjib Guha, Sarah Fischer, Anson Cheng, Gail V.W. Johnson, Keith Nehrke

**Affiliations:** 1 University of Rochester, Department of Anesthesiology & Perioperative Medicine, Rochester, NY; 2 University of Rochester, Department of Medicine, Nephrology Division, Rochester, NY

**Figure 1. Activation of the C. elegans mitochondrial unfolded protein response (UPRmt) in the PLM touch neuron by a tau mutant mimicking phosphorylation at T231:**
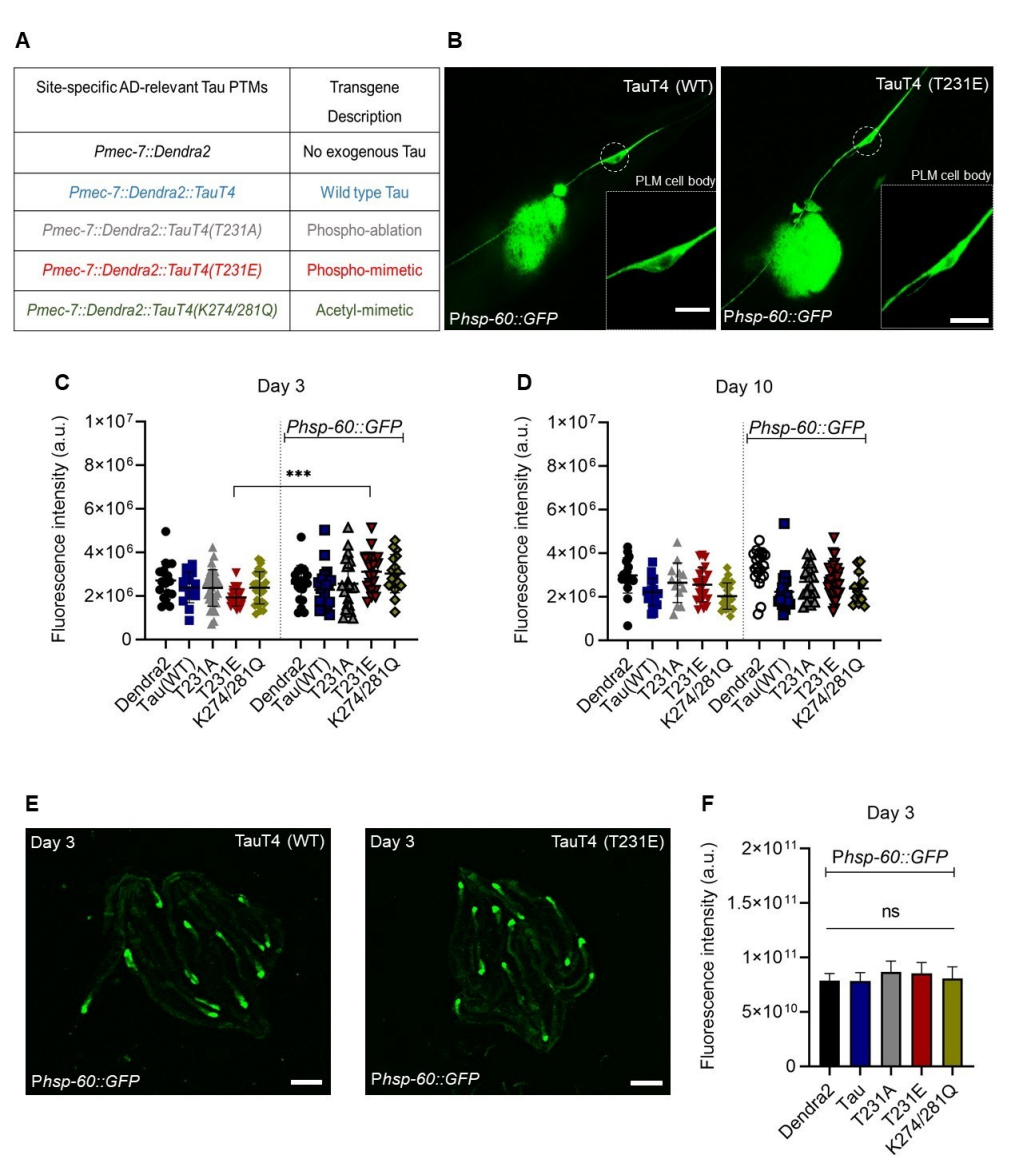
(A) A list of the strains used in this study. All transgenes code for translational fusions between TauT4 and the photo-convertible protein Dendra2, driven by the *mec-7* promoter specifically in touch neurons. CRISPR-Cas9 gene editing was used to introduce phospho-ablation T231A, phospho-mimetic T231E and acetyl-mimetic K274/281Q mutations into the TauT4 ORF. For simplicity, these mutants will be referred to as T231A, T231E and K274/281Q. (B, C) Fluorescent images of a touch neuron from day 3 adult worms expressing single-copy transgenes coding for a Dendra2::TauT4 translational fusion and the T231E mutant. Dashed circles indicate the location of the PLM cell body, shown magnified in the inset. Scale bar, 0.5 µm. Note, the blob fluorescence is coming from the hsp-60 expression tagged with GFP in the posterior intestine. (C, D) Quantification of the PLM cell body fluorescence for strains listed in panel A, at days 3 and 10 of adulthood. Data are the mean ± SD from two independent technical replicates. Individual data points demarcate values from single PLM cells from separate animals (N = 25 ± 5). Statistical analysis was by two-way ANOVA with Tukey’s post hoc test, with *** P< 0.001 when comparing bracketed samples. Note, the left side bar columns refer to the quantification of the fluorescence coming from the transgenic strains carrying Dendra2 reporter alone, whereas the right side ones refer to the strains carrying both Dendra2 and hsp-60 reporters. (E) Representative fluorescent images of transgenic worms expressing the integrated UPR^mt^ reporter P*hsp-60::*GFP and single-copy MosSCI insertions containing either wild type TauT4 or T231E. Scale bar, 0.5 mm. (F) Quantification of the fluorescence signal intensity in the posterior intestinal region from the strains listed in panel A. Data are the mean ± SD (N=20 animals from two independent biological replicates). ns denotes not significant, as calculated via one-way ANOVA followed by Tukey’s multiple comparisons test.

## Description

Alzheimer’s disease (AD) is the most common progressive neurodegenerative disorder (Selkoe *et al.*, 2001). One of the key pathological hallmarks of AD is neurofibrillary tangles (NFTs), which are primarily composed of abnormally modified tau (Avila *et al.*, 2004). Tau isolated from AD brain exhibits a number of posttranslational modifications (PTMs); including increases in phosphorylation and acetylation at specific epitopes that likely impair its function (Neddens *et al.*, 2018). Phosphorylation of tau at threonine 231 (T231) causes significant changes in tau structure, thus impairing microtubule binding (Mi *et al.*, 2006; Quintanilla *et al.*, 2014). In addition, increased expression of tau acetylated at Lysine 274 (K274) and Lysine 281 (K281) appears to result in mislocalization of tau, destabilization of the cytoskeleton in the axon initial segment, and synaptic dysfunction (Tracy *et al.*, 2016). Even though it is widely accepted that tau with aberrant PTMs facilitate neurodegeneration, the precise cellular mechanisms remain unknown. Mounting evidence suggests selective pathological tau species compromise mitochondrial biology (Reddy *et al.*, 2011; Cummins *et al.*, 2019). Understanding the molecular mechanisms through which this occurs will help to delineate the role tau plays in AD. Mitochondrial quality control mechanisms play a key role in restoring cellular homeostasis following stress. In addition, these mechanisms promote mitochondrial recycling through a form of selective autophagy termed mitophagy, are an attractive target to consider in the context of AD (Kerr *et al.*, 2017).

To interrogate the effects of pathologic PTMs in *Caenorhabditis elegans* model system, CRISPR-Cas9 gene editing (Paix *et al.*, 2015) was used to introduce a disease-associated phosphorylation mimicking (T→E) or a non-phosphorylatable (T→A) mutation at the T231 position of the wild-type TauT4 isoform, or alternatively acetylation mimicking (K→Q) mutations at the K274 and K281 positions, as listed in Fig. 1A. Overall, our results clearly demonstrated that the induction of mitophagy occurring in response to the mitochondrial toxin paraquat was entirely suppressed by expression of the T231E and K274/281 mutants, but not of wild type TauT4 itself (Guha *et al.*, 2020).

One intriguing possibility is that these two TauT4 PTM-mimetic mutants might induce a mild adaptive stress response during development that dampens subsequent responsiveness following overt stress. In fact, it has been well documented that many signaling pathways that sense stress have feedback loops to suppress their sustained activation (Hotamisligil *et al.*, 2016), supporting at least plausibility. The mitochondrial unfolded protein response (UPR^mt^) is one such pathway. The UPR^mt^ is a surveillance pathway that was first identified in mammals, but has been best characterized genetically in the nematode *C. elegans* (Haynes *et al.*, 2007; Melber *et al.*, 2018). Induction of UPR^mt ^initiates a mitochondria-nuclear signaling axis that protects against stresses caused by respiratory chain deficits, excessive reactive oxygen species, unfolded proteins, and pathologic bacteria (Rolland *et al.*, 2019; Peña *et al.*, 2016). In *C. elegans,* HSP-60 is a matrix-localized mitochondrial molecular chaperone whose expression has been widely used as a surrogate for activation of the UPR^mt^ (Bennett *et al.*, 2014; Benedetti *et al.*, 2006). The expression of an integrated transgene where GFP is driven by the *hsp-60* promoter is restricted under basal condition to the posterior cells of the intestine, but following induction of the UPR^mt^ is expressed more widely throughout the body (Gitschlag *et al.*, 2016). Here, we asked whether the expression of P*hsp-60::*GFP in touch neurons is influenced by different PTMs of tau.

The observation that measurable green fluorescence could be observed in the cell body of PLM touch neurons even in the absence of the P*hsp-60::*GFP transgene(Fig. 1B and 1C), is consistent with the fluorescent spectra of the photo-convertible Dendra2 tag, which is present in all of the transgenic strains, albeit at single copy. However, in strains containing the P*hsp-60::*GFPtransgene, only the phospho-mimetic T231E mutant exhibited a significant increase in PLM cell body fluorescence (Fig. 1C). The magnitude of the response is small, but this result is consistent with T231E causing mild mitochondrial stress and cell-autonomous activation of the UPR^mt^. Interestingly, UPR^mt^ activation in the K274/281Q acetyl-mimetic mutant, which like T231E caused neurodegeneration and an inability to trigger mitophagy in response to paraquat treatment (Guha *et al.*, 2020), failed to reach significance (*p = 0.1*), but also had increased scatter in the baseline signal (Fig. 1C).

One advantage of the worm model with its short three weeks lifespan is the ability to relate longitudinal effects over time to aging. In this context we note that the increased P*hsp-60::*GFP expression in T231E was limited to day 3 of adulthood, and that the difference between fluorescence in the absence versus the presence of the P*hsp-60::*GFP transgene was not significant at day 10 in any of the strains (Fig. 1D). While this may have been due to the limited magnitude of the response and a higher baseline fluorescence in older animals (Fig. 1D), it is also possible that it reflects the fact that the UPR^mt ^is thought to be restricted to young animals (Wu *et al.*, 2018).

Another distinct advantage of the worm model is the ability to discern between cell autonomous and cell non-autonomous activation of stress signaling pathways, a mode of activation relevant to both the UPR^mt^ (Durieux *et al.*, 2011) and the more widely studied UPR^er^ in worms (Frakes *et al.*, 2017). However we were unable to detect any change in intestinal P*hsp-60::*GFP expression across the entire repertoire of tau mutants (Fig. 1E, F), suggesting that the increased expression we observed in the T231E mutant was limited to a cell autonomous effect.

Our results clearly demonstrate that wild type tau expressed at single copy level does not activate the UPR^mt^ in worm touch neurons, but that a mutation mimicking a pathological PTM that has been associated with AD causes subtle UPR^mt^ activation in young adult worms. We hypothesize that this chronic low-level activation could suppress subsequent responses to mitochondrial stress.

## Methods

***C. elegans* strains growth and maintenance**

Nematodes were maintained at 20^0^C on Nematode Growth Media (NGM) plates made with Bacto Agar (BD Biosciences). The plates were seeded with live *E. coli* OP50-1 bacterial strain (cultured overnight at 37^o^C at 220 rpm) and allowed to grow overnight. For experimental assays, after synchronization by standard procedure with sodium hypochlorite, 4^th^ larval stage (L4) hermaphrodites (characterized by the appearance of a “Christmas tree vulva”) were selected and moved to test plates. The day after moving was considered adult day 1, and animals were assayed on day 3 and day 10. Animals were transferred daily to avoid mixed population until they stop laying eggs.

**Fluorescent imaging assay**

Animals were mounted on 2% agarose pads on glass slides and immobilized with 1 mM tetramisole hydrochloride before imaging. Imaging was performed using a Nikon Eclipse inverted microscope coupled to a six channel LED light source (Intelligent Imaging Innovation, Denver, CO), an ORCA-Flash4.0 V2 Digital CMOS camera (Hamamatsu Photonics, Bridgewater Township, NJ) and Slidebook6 software (Intelligent Imaging Innovation, Denver, CO). All images were acquired under the same exposure conditions and each experiment was imaged in one session. The PLM cell body was identified by their position toward the posterior of the animal, near the tail and was focused with a 100x oil immersion lens under visible light using DIC contrast. 600-nm+ emissions were captured first following excitation at 440-nm, keeping light intensity and exposure times constant between images. Images were quantified using ImageJ software by selecting the ROI, measuring the mean intensity for green channels and subtracting the background intensity. N.B. – We only quantified PLM cell body fluorescence, not the ALM fluorescence because it might interfere with the intestinal gut fluorescence, giving us a faulty reading.

**Statistical Analysis**

All statistical analyses were conducted using Prism 8.0 (GraphPad Software), with alpha-error level of *p < 0.05* considered to be significant. Data were averaged and represented as mean ± standard deviation (mean ± SD). In general, group differences were analyzed with either one-way or two-way ANOVA depending upon the variables. The sample sizes were based on those found previously in the laboratory to provide appropriate power for discerning phenotypic differences among genotypes.

## Reagents

SJ4058: *zcIs9* [hsp-60::GFP + lin-15(+)]. This is a stable transgenic line with low basal GFP expression, mainly in the tail, observed from L1 to adult animals. Transgenic strains include the following: KWN169, *rnySi26 [Pmec-7::Dendra2; unc-119+] II*; KWN167, *rnySi24 [Pmec-7::Dendra2::Tau-T4; unc-119+] II*. KWN788 *rnySi51* [Tau-T4 (T231A) *rnySi24] II, KWN789 rnySi52 [Tau-T4 (T231E) *rnySi24] II, KWN790 rnySi53 [Tau-T4 (K274Q; K281Q) *rnySi24] II. For crossing tau MosSCI strains into hsp-60 reporter strain, Dendra2 fluorescent was used to guide selection of homozygous mutants, and PCR genotyping was used to confirm homozygosity with primers specific to the ttTi5605 loci, including:

MosSCI ttTi5605-F, 5’GTTTTTGATTGCGTGCGTTA3’

MosSCI ttTi5605-R, 5’ACATGCTTCGTGCAAAACAG3’

MosSCI ttTi5605 insert-F, 5’CATCCCGGTTTCTGTCAAAT3’
